# Injury and differentiation following inhibition of mitochondrial respiratory chain complex IV in rat oligodendrocytes

**DOI:** 10.1002/glia.21052

**Published:** 2010-11-15

**Authors:** Iryna Ziabreva, Graham Campbell, Julia Rist, Jessica Zambonin, Joanna Rorbach, Mateusz M Wydro, Hans Lassmann, Robin J M Franklin, Don Mahad

**Affiliations:** 1 The Mitochondrial Research Group, Newcastle UniversityFramlington Place, Newcastle upon Tyne, United Kingdom; 2 MRC Centre for Stem Cell Biology and Regenerative Medicine and Department of Veterinary Medicine, University of CambridgeCambridge, United Kingdom; 3 Department of Neuroimmunology, Center for Brain Research, Medical University ViennaVienna, Austria

**Keywords:** mitochondria, oligodendrocytes, cytochrome c oxidase

## Abstract

Oligodendrocyte lineage cells are susceptible to a variety of insults including hypoxia, excitotoxicity, and reactive oxygen species. Demyelination is a well-recognized feature of several CNS disorders including multiple sclerosis, white matter strokes, progressive multifocal leukoencephalopathy, and disorders due to mitochondrial DNA mutations. Although mitochondria have been implicated in the demise of oligodendrocyte lineage cells, the consequences of mitochondrial respiratory chain defects have not been examined. We determine the *in vitro* impact of established inhibitors of mitochondrial respiratory chain complex IV or cytochrome c oxidase on oligodendrocyte progenitor cells (OPCs) and mature oligodendrocytes as well as on differentiation capacity of OPCs from P0 rat. Injury to mature oligodendrocytes following complex IV inhibition was significantly greater than to OPCs, judged by cell detachment and mitochondrial membrane potential (MMP) changes, although viability of cells that remained attached was not compromised. Active mitochondria were abundant in processes of differentiated oligodendrocytes and MMP was significantly greater in differentiated oligodendrocytes than OPCs. MMP dissipated following complex IV inhibition in oligodendrocytes. Furthermore, complex IV inhibition impaired process formation within oligodendrocyte lineage cells. Injury to and impaired process formation of oligodendrocytes following complex IV inhibition has potentially important implications for the pathogenesis and repair of CNS myelin disorders. © 2010 Wiley-Liss, Inc.

## INTRODUCTION

Demyelination is a cardinal neuropathological feature of several central nervous system (CNS) disorders including multiple sclerosis (MS), white matter strokes (WMS), progressive multifocal leukoencephalopathy (PML), periventricular leukomalacia (PVL), and certain primary mitochondrial disorders due to mitochondrial DNA (mtDNA) mutations (Aboul-Enein et al., 2003; Barnett and Prineas, 2004; Betts et al., 2004; Gendelman et al., 1985; Lucchinetti et al., 2000; Tanji et al., 2001). Oligodendrocytes, the myelin forming cells in the CNS, are derived from oligodendrocyte progenitor cells (OPCs). OPCs persist in the CNS throughout adulthood and are responsible for regeneration (remyelination) in demyelinating diseases (ffrench-Constant and Raff, 1986; Franklin and ffrench-Constant, 2008; Levine et al., 2001). Oligodendrocyte lineage cells are particularly susceptible to insults such as hypoxia, excitotoxicity, reactive oxygen, and nitrogen species and cytokines (Cannella and Raine, 2004; Merrill and Scolding, 1999; Smith et al., 1999). The demise of oligodendrocyte lineage cells following a number of insults has been shown to involve mitochondria (Baud et al., 2004a, b; Fragoso et al., 2004; Itoh et al., 2000; Schoenfeld et al., 2009; Scurlock and Dawson, 1999).

Mitochondria are not only important for ATP production but also involved in apoptosis and calcium handling (Degterev and Yuan, 2008; Taylor et al., 2008). The mitochondrial respiratory chain consists of five complexes (complex I–complex V) made up of multiple subunits. Uniquely, mitochondria harbor the only non-nuclear DNA, mtDNA, which encodes four of the five complexes including complex IV. Complex IV or cytochrome c oxidase (COX), the terminal complex of electron transport chain, is where over 90% of oxygen is consumed (DiMauro and Schon, 2003). Complex IV is frequently affected in primary mitochondrial disorders (Betts et al., 2004; Tanji et al., 2001). In MS, complex I and complex IV defects have been reported in chronic white matter and acute Balo's type lesions, respectively (Lu et al., 2000; Mahad et al., 2008). Complex IV defects in acute MS lesions, based on the loss of the main catalytic subunit (COX-I), affected oligodendrocytes, astrocytes, and axons (Mahad et al., 2008). Although well-established complex IV inhibitors, sodium azide and potassium cyanide, have been used to induce chemical hypoxia *in vitro* and *in vivo* the consequences of complex IV inhibition in oligodendrocyte lineage cells have not been determined (Bennett et al., 1996; Keilin and Hartree, 1945; Marino et al., 2007; Micu et al., 2006). A recent study identified differentiating oligodendrocytes as more susceptible to inhibition of complex I by rotenone than OPCs (Schoenfeld et al., 2009). Furthermore, complex I inhibition at levels that did not compromise cell viability blocked OPC differentiation (Schoenfeld et al., 2009).

In this study, we investigated the impact of complex IV inhibition on oligodendrocyte lineage cells from P0 rat and on the capacity of OPCs to differentiate*, in vitro*. Injury mediated by complex IV inhibitors in mature oligodendrocytes (identified by MBP expression) was greater than in OPCs (identified by PDGFRA or NG2 expression). Mitochondria were abundant and mitochondrial membrane potential (MMP), an electrochemical gradient generated and maintained across the inner mitochondrial membrane by proton pumps including complex IV, was significantly greater in processes of differentiated oligodendrocytes compared to OPCs. The formation of oligodendrocyte processes was notably impaired when complex IV was inhibited throughout or briefly at onset of OPC differentiation. Thus, mitochondrial respiratory chain dysfunction both injures mature oligodendrocytes and impairs differentiation of OPCs from P0 rat. These affects have potentially important implications for demyelinating disorders, including PVL and MS.

## MATERIALS AND METHODS

### Cell Culture

Mixed glial cultures were prepared according to the method of McCarthy and DeVillis (McCarthy and de Vellis, 1980) with minor modifications. Briefly, the meninges, midbrain and olfactory bulbs were removed, and dissociated rat neonatal cortices (postnatal Day 0 or P0) cut thoroughly and incubated at 37°C for 1 h in MEM containing 4% Papain, 1% of 4 mg/mL DNase I Type IV and 1% of 24 mg/mL L-cystein. Dissociated cells were plated on poly-D-lysine coated tissue culture flasks and grown at 37°C with 7.5% CO_2_ for 10 days in DMEM +10% FCS +1%Pen/Strep. OPCs were derived from mixed glial cultures containing OPCs and microglia grown on astrocyte monolayer by shaking the flask first for 1 h at 250 rpm and then, after replacing cell culture medium, overnight for a minimum of 18 h at 450 rpm. Microglia were removed by differential adhesion method. OPCs were seeded into PDL-coated 8-well chamber slides at a density of 60,000 cells/mL (15,000 cells per chamber) and maintained in SATO medium. For the first 2 days, 10 ng/mL PDGF and 10 ng/mL FGF were added for proliferation and afterwards the medium was replaced by SATO with 0.5% FCS for differentiation. Cells remained in SATO with 0.5% FCS for the 5-day-period of differentiation with replacement of 25% of the medium (100 μL) on the 4th day. Cell viability was determined using Trypan blue and ethidium homodimer extrusion methods as well as TUNEL staining. The sublethal range of sodium azide and potassium cyanide concentrations (1–100 μM) was determined based on greater than 90% of oligodendrocyte lineage that remained attached in the chamber slides being viable judged by Trypan blue and ethidium homodimer extrusion and TUNEL staining.

### Cytospins

Cytospins were used to capture the cells that detach from the chamber slides of control and sodium azide treated chambers and remain suspended in the supernatant. The supernatant was transferred from chambers into cytofunnel with filter card attached to a cytoslide and placed into Cytocentrifuge. The density of detached cells in the supernatant was determined using a hemocytometer prior to centrifuging for 4 min at 1200 rpm. The slides were dried for 1 h prior to immunocytochemical staining (Table 1). The percentage of cells expressing caspase 9 and MBP or PDGFRA was used for quantitation rather than cell density as cytospins cause cell loss and non-uniform distribution of cells.

**Table 1:** Details of Antibodies Used for Immunohistochemistry and Immunocytochemistry

Antigen	Target	Antibody	Source
Olig2	Transcription factor Olig2	Rabbit IgG	Chemicon
MBP (SMI-94R)	Myelin basic protein	Mouse IgG_1_	Covance
PDGFRA	Platelet-derived growth factor receptor alpha	Mouse IgG_1_	R&D Systems
NG2	NG2 chondroitin sulfate proteoglycan	Rabbit IgG	Chemicon
Caspase 9	Cleaved caspase 9 (asp353)	Rabbit IgG	Cell Signaling Technology
AIF	Apoptosis inducing factor	Rabbit IgG	Cell Signaling Technology
GFAP	Glial Fibrillary Acidic Protein	Rabbit IgG	DAKO
ED1	Macrosialin	Mouse IgG_1_	AbD Serotec
Ox42	CD11b/c	Mouse IgG_2a_	Abcam

### Immunocytochemistry

For immunocytochemical labeling of P0 rat oligodendrocyte lineage cells (Table 1) in culture, 8 well chamber slides were fixed in PBS containing 2% paraformaldehyde for 10 min. The fixed cells were incubated in normal goat serum to prevent non-specific binding of antibodies. The secondary antibodies directly conjugated with fluorochromes (Alexa fluor 488, 568, or 633) were used from molecular probes. The appropriate controls were performed to exclude cross-reactivity and non-specific binding. OPCs were identified based on expression of PDGFRA and NG2 whereas mature oligodendrocytes expressed MBP.

### Mitochondrial Isolation and Spectrophotometric Measurement of Complex IV and Citrate Synthase Activity

Cells were washed briefly with PBS and detached from Petri dishes using TripLE™ Express. SATO with 0.5% FCS was added to detached oligodendrocyte lineage cells before centrifuging. The cell pellet was washed twice in sterile PBS, resuspended in 1 mL medium B containing 250 mM Sucrose, 2 mM HEPES, and 0.1 mM EGTA and kept on ice. The resuspended cells were transferred into a glass homogenizer tube (2 mL Wheaton-smooth surface) and gently disrupted with a Teflon-coated tissue homogenizer. The homogenate was centrifuged for 10 min at 2200 rpm at 4°C. The pellet was resuspended before spectrophotometric analysis of complex IV and citrate synthase activity as previously described (Kirby et al., 2007).

### Respirometry

For respirometry, differentiated cells with or without exposure to complex IV inhibitor were washed briefly with PBS, dissociated from plastic using TripLE™ Express and resuspended in SATO with 0.5% FCS. High-resolution respirometry was performed at 37°C using an Oroboros Oxygraph-2K (Oroboros Instruments, Innsbruck, Austria). Approximately 0.5 × 10^6^ cells were added to the chamber in 2 mL of respiration buffer (glucose free DMEM, 10% FCS, 0.11 mg/mL Na pyruvate, 0.9 mg/mL galactose), and routine respiratory oxygen flux was established over 10 min with automatic deduction of background flux using the online DatLab software (Oroboros Instruments, Innsbruck, Austria). Cell counts were made using a small aliquot of cells at the end of the experiment, and rates were then normalized to pmol of oxygen consumed per 10^6^ cells per unit of time.

### Measurement of Mitochondrial Membrane Potential

MMP of P0 rat oligodendrocyte lineage cells was measured using monomeric JC-1 (5,5′,6,6′-tetrachloro-1,1′,3, 3′-tetraethylbenzimidazolyl-carbocyanine iodide), which aggregates in mitochondria depending on the MMP, at a concentration of 10 μg/mL for 20 min. The ratio of densitometric values for red (590 nm) and green (510–527 nm) fluorescence was used as an indicator of MMP as previously described (Baud et al., 2004b). Differentiated oligodendrocytes (5 days) were compared with OPCs (proliferated without differentiation for 5 days) as immunolabeling to identity oligodendrocytes interfered with MMP and its measurement.

### Measurement of Mitochondrial Reactive Oxygen Species

**Figure 1:**
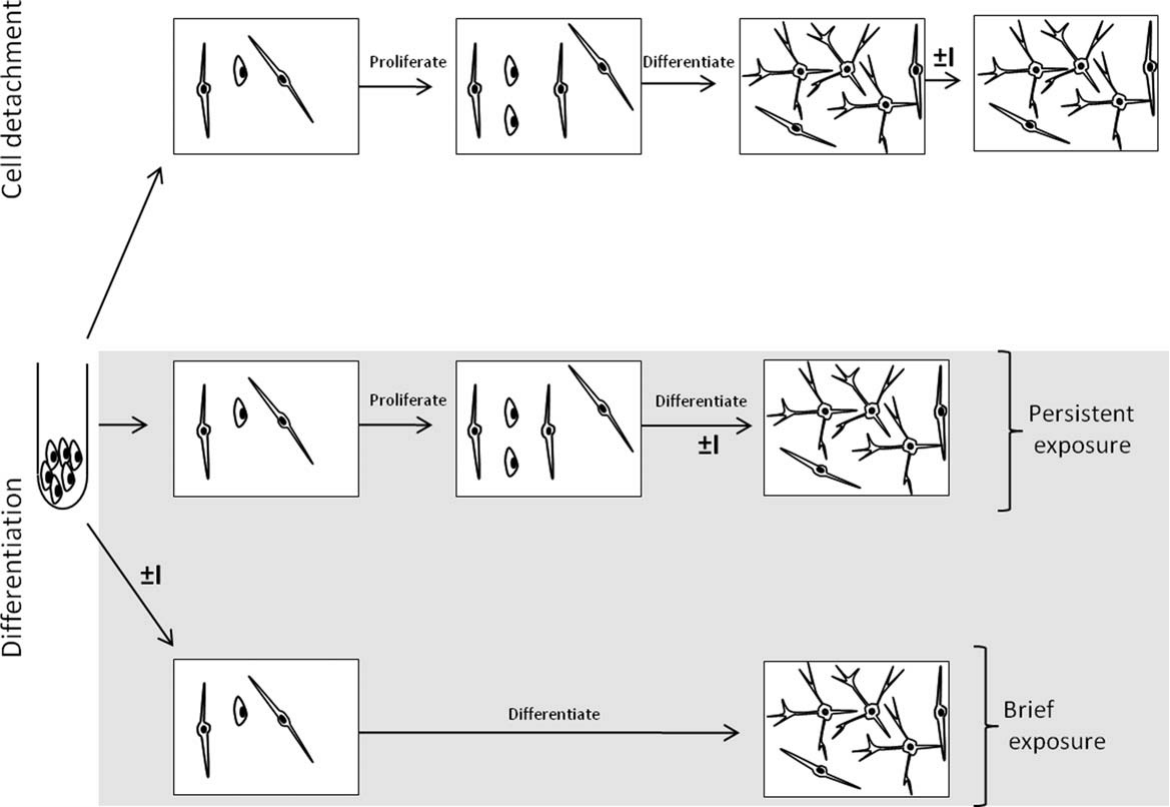
Experimental timeline. To investigate injury to and detachment of cells following complex IV inhibition, P0 rat OPCs were proliferated for 2 days with PDGF and FGF and then differentiated for 5 days prior to exposure to sodium azide (1, 10, and 100 μM) or potassium cyanide (10 μM and 100 μM). The differentiation capacity of OPCs was assessed by either persistent or brief exposure to complex IV inhibitor at concentrations that did not reduce the number of OPCs (up to 10 μM). In persistent exposure experiments, complex IV inhibitor was added at the onset of OPC differentiation and kept throughout differentiation (persistent exposure) in the media, 25% of which was replaced on Day 4. To rule out the possibility that oligodendrocytes differentiate and then lost rather than fail to differentiate in persistent exposure experiments, OPCs were briefly exposed to sodium azide for 10 min and then washed prior to differentiation (brief exposure). The control samples, appropriate for the time points analyzed, were not exposed to the inhibitor. I: complex IV inhibitor (sodium azide or potassium cyanide).

Differentiated oligodendrocytes and proliferated OPCs from P0 rat (Fig. .1), with or without exposure to complex IV inhibitors (1 μM, 10 μM, and 100 μM for 15 min, 3 h, and 36 h), were resuspended using TripLE™ Express (GIBCO). MitoSOX (Molecular Probes) prepared in DMSO was added to resuspended cells, washed in PBS, for 10 min at 37°C. The cells were washed and analyzed using a FACScan flow cytometer (Becton Dickinson, Oxford, UK, equipped with 488 nm Argon laser). Experiments were performed on three independent occasions with measurements based on 10,000 events.

### Assessment of Cell Viability and Morphology

To assess cell viability we identified cells that failed to extrude Trypan blue and ethidium homodimer. After removing media and washing with PBS, the cells were incubated for 5 min in 50% Trypan blue or ethidium homodimer followed by brief wash with PBS. All cells that were stained blue or contained red fluorescence were counted in 20 adjacent ×40 fields of view per chamber. The assessment of TUNEL positive cells was performed using *In Situ* Cell Death Detection Kit, TMR red (Roche Diagnostics) following brief fixation in 4% paraformaldehyde in PBS pH 7.4 and immunocytochemical labeling of oligodendrocyte lineage cells. Following immunocytochemistry, the cells on slides were washed three times for 5 min in PBS and incubated in permeabilization solution [freshly prepared 0.1% sodium citrate (BDH) and 0.1% Triton X-100 (Sigma)] for 3 min on ice. Following three washing steps, the TUNEL reaction mixture with enzyme and label solutions was applied to the slides according to manufacturer's instructions. Nuclear staining was performed using DAPI. Three independent experiments, each in triplicate chambers, were performed for controls and different concentrations of complex IV inhibitors. Following differentiation of OPCs the morphology of mature oligodendrocytes expressing MBP were graded. Cells without processes (category I), with primary or simple processes (category II), with secondary or complex processes (category III) and with myelin membranes based on the presence of continuous myelin staining between processes (category IV) were identified.

### Microscopy and Quantitation

Immunocytochemical and immunohistochemical stains were analyzed using Zeiss Axiovert and Zeiss Axioplan microscopes (Germany), respectively. For ICC (attached cells in slides and detached cells in cytospins), 10 fields (×20) were randomly chosen per slide or cytospin and FITC, Rhodamine and DAPI channels were imaged using an Axiocam mRM (Zeiss). The cells of interest were manually counted per field (10 fields per chamber in triplicates) and an average obtained per chamber or cytospin for each experiment.

### Statistics

Significant differences between multiple groups were analyzed using one-way ANOVA and differences between two groups was determined using Student's *t* test.

## RESULTS

### Mature Oligodendrocytes More Susceptible to Injury than Oligodendrocyte Progenitor Cells Following Complex IV Inhibition

**Figure 2:**
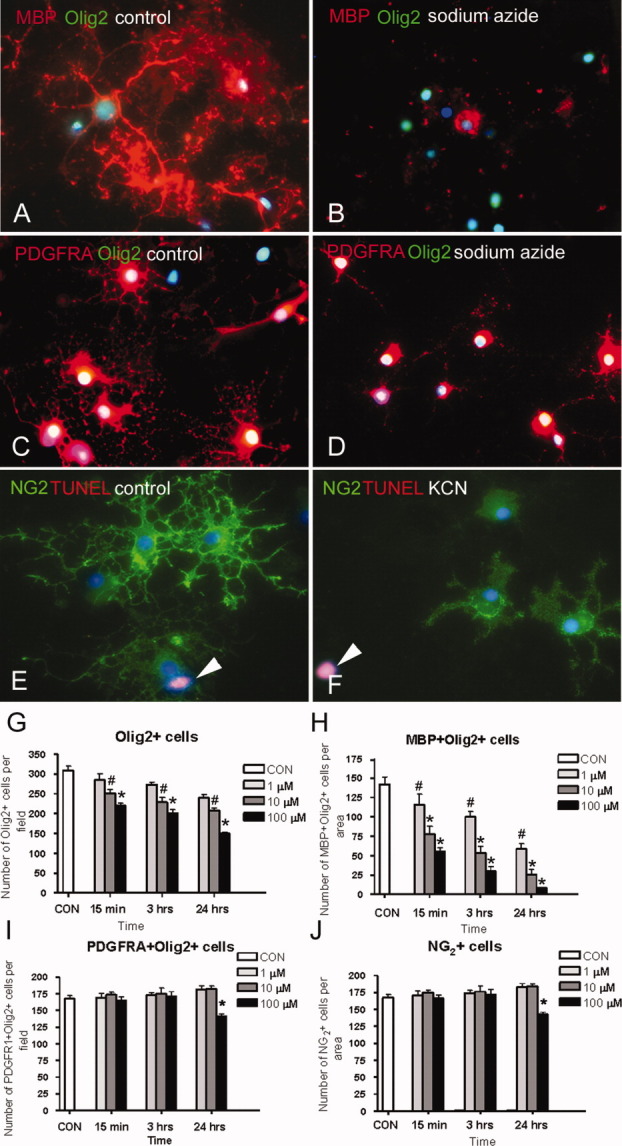
Injury to oligodendrocyte lineage cells from P0 rat following complex IV inhibition. **A**, **C**, and **E**: There were mature oligodendrocytes expressing MBP (a) and OPCs expressing PDGFRA (c) or NG2 (e) in oligodendrocyte cultures, derived from purified OPCs proliferated for 2 days and differentiated for 5 days. **B**, **D**, and **F**: Injury to processes of oligodendrocyte lineage cells and loss of mature oligodendrocytes (b) were the most striking changes observed following inhibition of complex IV. Images show changes following 100 μM of sodium azide for 3 h (b and d) and 100 μM of potassium cyanide for 3 h. TUNEL positive nuclei (e and f, arrowheads) were detected on average in 13.7% of oligodendrocyte lineage cells, which did not change significantly following complex IV inhibition. **G**–**J**: The quantitation of oligodendrocyte lineage cells remaining attached in the chamber slides showed a concentration dependent reduction in absolute number of Olig2+ nuclei (g). Loss of Olig2+ cells was confirmed by DAPI staining. Mature oligodendrocytes were injured and detached following complex IV inhibition in a dose dependent manner (h). In contrast, OPCs (PDGFRA+ or NG2+) remained attached to the chamber slides and the numbers were not effected by complex IV inhibition, except with 100 μM sodium azide at 24 h (i and j). The experiments, controls, and different concentrations of inhibitors in triplicate chambers, were performed on 10 separate occasions from six different litters. *P < 0.001. ^#^*P* < 0.01. [Color figure can be viewed in the online issue, which is available at wileyonlinelibrary.com.]

The exposure of oligodendrocyte lineage cells from P0 rat to 1 μM, 10 μM, and 100 μM of sodium azide for 3 h decreased complex IV activity of mitochondria isolated from oligodendrocytes by 38.6% (mild), 68.7% (moderate), and 83.9% (severe), respectively, compared with control samples when normalized using citrate synthase. In differentiated cultures, 93.7% of DAPI positive nuclei, on average, expressed Olig2 and oligodendrocyte lineage cells were then characterized based on MBP or PDGFRA and NG2 labeling (Fig. .2a–f). Less than 5% of cells were astrocytes based on expression of GFAP and microglia were not detected in differentiated cultures using ED1 and Ox42 staining.

Complex IV inhibition led to a striking loss of processes in P0 rat oligodendrocyte lineage cells (Fig. .2a–f). The extent of injury to oligodendrocytes was reflected by a decrease in the absolute number of cells following inhibition of complex IV (Fig. .2g). The cell loss was confirmed by the reduction of DAPI nuclei. Nearly all the cell loss following complex IV inhibition was due to detachment of mature MBP+ oligodendrocytes (Fig. .2h). Mature oligodendrocytes remaining on the slides decreased significantly in a dose dependent manner when exposed to complex IV inhibitor whereas (Fig. .2i,j) the number of OPCs identified by PDGFRA or NG2 was unchanged, except with the highest concentration of the inhibitor at 24 h where both OPC and oligodendrocyte numbers were reduced (Fig. .2g–j). To our surprise 92.8% of all cells that remained attached to chamber slides extruded Trypan blue or ethidium homodimer and 86.3 and 93.7% of cells on the chamber slides did not contain a TUNEL positive or fragmented nucleus, following severe complex IV inhibition. Potassium cyanide also caused a similar degree of complex IV inhibition (Cooper and Brown, 2008) and preferential loss of MBP expressing cells compared with OPCs (Supp. Info. Fig. 1).

### Oligodendrocytes Detach, Express Caspase 9, and Translocate Apoptosis Inducible Factor to Nucleus Following Complex IV Inhibition

**Figure 3:**
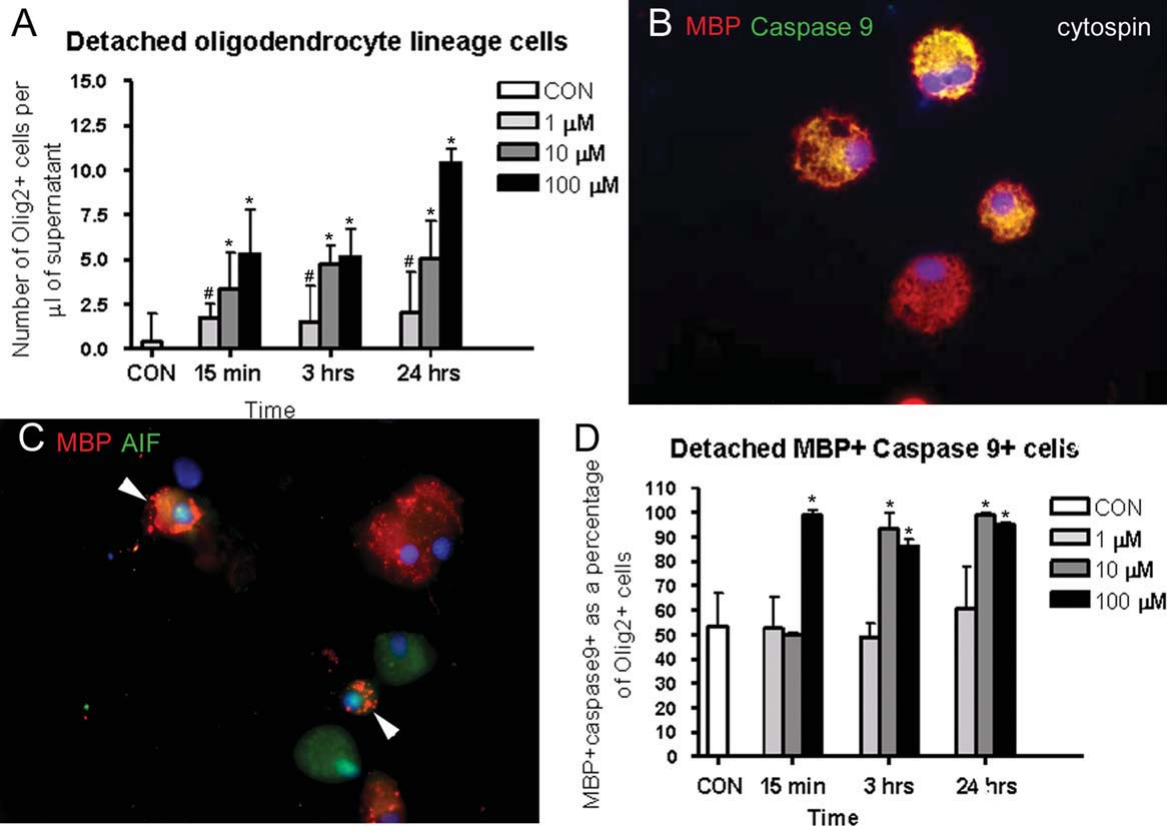
Detached oligodendrocytes in supernatant reflect cell loss following complex IV inhibition and express caspase 9 and translocate AIF to nucleus. **A**: The density of oligodendrocyte lineage cells in the supernatant, later captured on cytospins, increases in a dose dependent manner following exposure to sodium azide, reflecting the extent of cell loss in chamber slides (Fig. .2e). A density of 10 cells per μL in 400 μL of supernatant from one chamber represents detachment of 32% of cells. **B** and **C**: Detached MBP expressing cells captured on cytospins express caspase 9 (b) and show evidence of nuclear translocation of AIF (c, arrowheads). AIF positive cells without MBP expression (arrow) are most likely detached OPCs. Co-staining of caspase 9 and AIF was not possible as both primary antibodies were raised in rabbit. **D**: The extent of caspase 9 expression in detached cells varies in a dose and time dependent manner, with the majority of MBP expressing cells expressing caspase 9 when exposed to 100 μM of sodium azide. Caspase 9 was not detected in approximately half of the detached MBP expressing cells in control chambers or following exposure to 1 μM of sodium azide. The experiments, controls, and different concentrations of inhibitors in triplicates, were performed on four occasions from different litters. *P < 0.001. ^#^*P* < 0.01. [Color figure can be viewed in the online issue, which is available at wileyonlinelibrary.com.]

To confirm that the cell loss following mitochondrial inhibition occurred prior to immunostaining we determined the cell density in supernatant following complex IV inhibition, which reflected oligodendrocyte lineage cell loss (Fig. .3a). The detached cells in cytospins expressed caspase 9 and there was evidence of nuclear translocation of AIF (Fig. .3b,c). The percentage of detached MBP+ cells expressing caspase 9 varied depending on the concentration of and duration of exposure to the inhibitor (Fig. .3d). Caspase 9 expression in detached cells was greater than in cells that remained attached to slides in controls and chambers with inhibitor (<8%). In detached OPCs (PDGFRA+ cells), caspase 9 and AIF expression did not change significantly following exposure to complex IV inhibitor with on average 8.6% of PDGFRA+ cells expressing caspase 9 and 48.5% showing nuclear translocation of AIF.

### Mitochondrial Membrane Potential Following Inhibition of Complex IV

**Figure 4:**
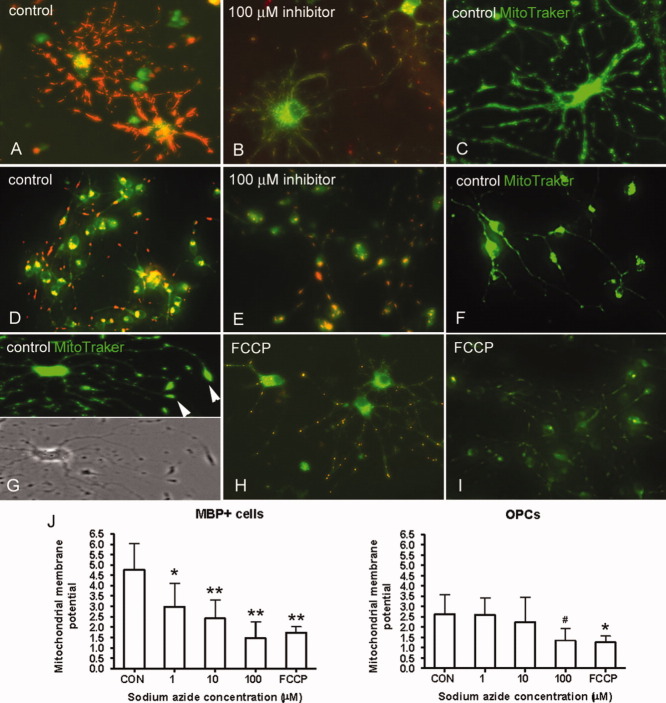
Mitochondrial membrane potential in live oligodendrocyte lineage cells from P0 rat. **A**–**I**: Mitochondrial membrane potential (MMP) was determined using JC-1. Oligodendrocyte processes in cultures differentiated for 5 days (a) contained strikingly more active mitochondria (red) than processes of OPCs proliferated without differentiation for 5 days (d). Exposure of differentiated oligodendrocytes and OPCs to complex IV inhibitor (shown following 10 μM of sodium azide exposure for 15 min) led to a notable dissipation of MMP (b and e). Non-aggregated JC-1 is shown by green fluorescence (a–b and d–e). The abundance of mitochondria, judged by JC-1 aggregation, in the processes of differentiated oligodendrocytes (c) relative to undifferentiated OPCs (f) was confirmed using MitoTraker green. The tips of differentiating oligodendrocytes, with bulbous morphology on brightfield image (g), were particularly notable for the presence of mitochondria (g, arrowheads). The mitochondrial membrane uncoupler, carbonylcyanide-*p*-trifluoromethoxyphenylhydrazone (FCCP), dissipated MMP in differentiated oligodendrocytes (h), and OPCs (i) as expected. **J**: Quantitation of MMP based on ratio of red to green fluorescence intensity showed the MMP to be much greater in differentiated oligodendrocytes compared with OPCs in controls. MMP decreased significantly in differentiated oligodendrocytes when exposed to 1 μM of sodium azide for 15 min whereas MMP in OPCs was less susceptible to low doses (1 μM and 10 μM) of complex IV inhibitor. *P = 0.001. **P < 0.001. ^#^*P* = 0.023. [Color figure can be viewed in the online issue, which is available at wileyonlinelibrary.com.]

Given the differential susceptibility of oligodendrocyte lineage cells from P0 rat to mitochondrial inhibitors, we determined the impact of complex IV inhibition on MMP (Fig. .4). Active mitochondria were abundant in the processes of differentiated oligodendrocytes whereas mitochondria were relatively sparse in processes of OPCs (Fig. .4a,d). The difference in the distribution of mitochondria between oligodendrocyte and OPC processes was confirmed using MitoTraker green (Fig. .4c,f). The exposure to complex IV inhibitor (100 μM of sodium azide for 15 min) led to a striking dissipation of MMP in both differentiated oligodendrocytes and OPCs (Fig. .4b,e). As expected, the uncoupler, carbonylcyanide-*p*-trifluoromethoxyphenylhydrazone (FCCP), caused a dramatic reduction in MMP within differentiated oligodendrocytes and OPCs (Fig. .4h,i).

Quantitation of MMP, based on the ratio between red and green fluorescence intensity of JC-1, showed the mitochondria in processes of differentiated oligodendrocytes with complex branch structure to be significantly more active than in processes of OPCs in control chambers (Fig. .4j). MMP in branches of differentiated oligodendrocytes with complex structure decreased significantly following exposure to 1 μM of complex IV inhibitor whereas 100 μM was required for a significant decrease in MMP within OPCs. Complex IV inhibitor (100 μM) dissipated MMP to similar extent to FCCP in oligodendrocyte lineage cells. The extent of MMP reduction as a percentage of baseline was greater in differentiated oligodendrocytes (71.3%) than OPCs (48.3%).

### Mitochondrial Superoxide Production Following Inhibition of Complex IV

Given the potential role of reactive oxygen species (ROS) in the vulnerability of oligodendrocyte lineage cells to a number of insults (see Supp. Info. Table 1), we measured mitochondrial superoxide in differentiated and then resuspended live oligodendrocytes as well as undifferentiated OPCs. Interestingly, superoxide levels in differentiated oligodendrocytes and OPCs exposed to sodium azide, at variable concentrations (1, 10, and 100 μM) and exposure times (15 min, 3 h, and 36 h), did not change compared with cells not exposed to inhibitors (Supp. Info. Fig. 2).

### Complex IV and Oligodendrocyte Progenitor Cell Proliferation

As OPCs from P0 rat withstand complex IV dysfunction better than their mature counterparts we investigated whether complex IV inhibition affected OPC proliferation. Interestingly, the exposure of OPCs to sodium azide did not affect PDGF and FGF mediated OPC proliferation over a 48 h period, judged by the absolute number of Olig2 expressing cells (Supp. Info. Table 2).

### Complex IV and Oligodendrocyte Progenitor Cell Differentiation

**Figure 5:**
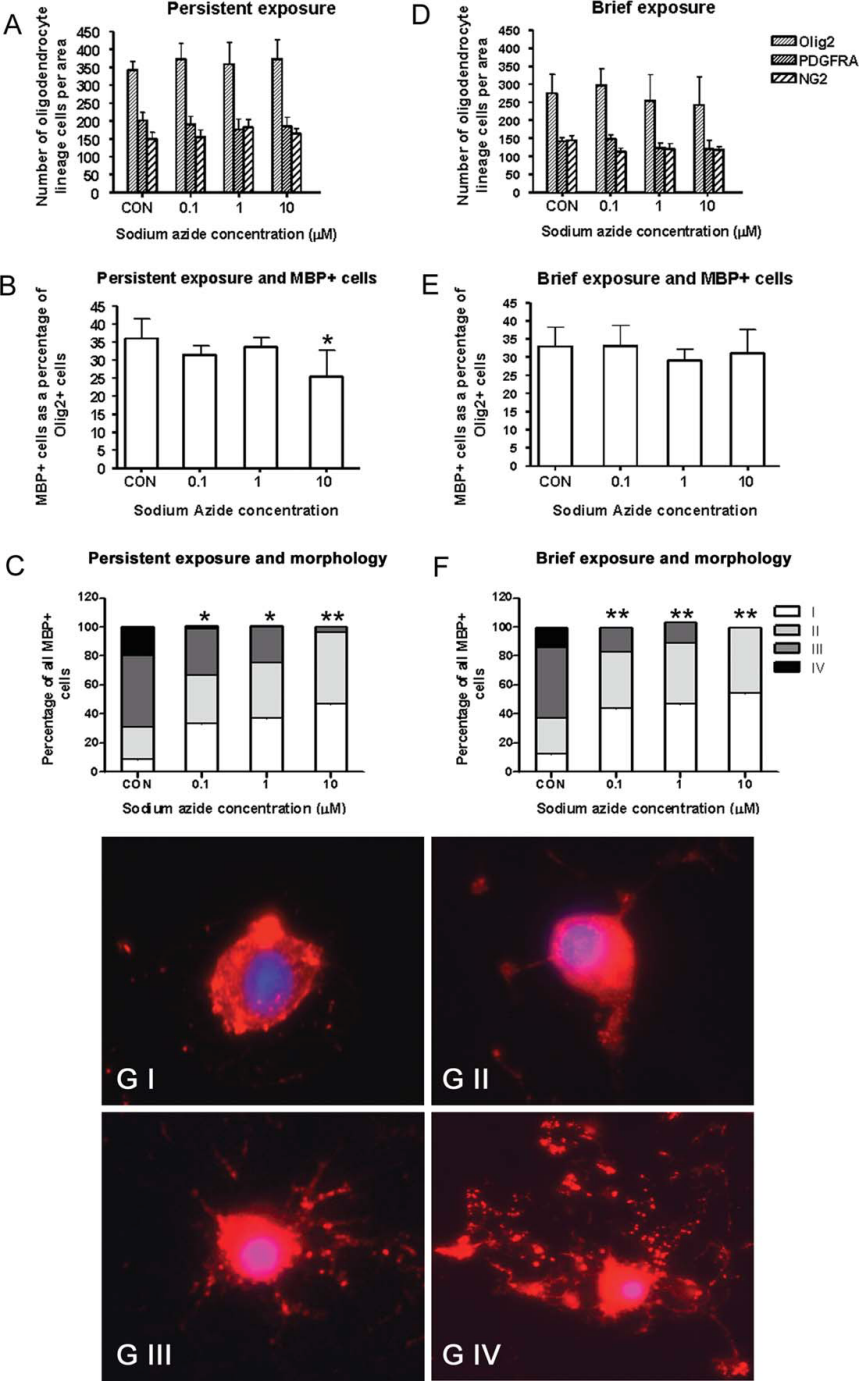
Density and morphology of mature oligodendrocytes when P0 rat OPCs were persistently or briefly exposed to complex IV inhibitor during or prior to differentiation. **A**–**C**: When proliferated OPCs were persistently exposed to sodium azide throughout the 5-day-period of differentiation (Fig. .1) at concentrations that did not effect the viability (up to 10 μM of sodium azide), the total number of oligodendrocyte lineage cells (MBP+, PDGFRA+, or NG2+) remaining in the chamber slides is similar compared with controls (a). With 10 μM of sodium azide there was a significant reduction in the percentage of MBP+ cells reflecting either impaired differentiation or injury to differentiated MBP+ cells (b). When mature oligodendrocytes were categorized into rounded (category I, GI), simple processes (category II, GII), complex processes (category III, GIII), or with MBP+ membrane (category IV, GIV) the proportion of MBP+ cells lacking processes or with simple processes was significantly greater when differentiated in the presence of complex IV inhibitor compared with controls (c). **D**–**F**: Brief exposure of OPCs to variable concentrations of sodium azide prior to differentiation (Fig. .1) led to similar number of oligodendrocyte lineage cells compared with controls (d). We did not detect a significant change in the number of MBP+ cells in brief exposure experiments (e). There was a significant increase in MBP expressing cells without processes or with simple processes in brief exposure experiments (f). The experiments, controls, and different concentrations of inhibitors in triplicate chambers, were performed on eight occasions from different litters. *P < 0.01. **P < 0.001. [Color figure can be viewed in the online issue, which is available at wileyonlinelibrary.com.]

As the next step we determine the impact of mitochondrial injury on differentiation capacity of P0 rat OPCs using complex IV inhibitors at concentrations (up to 10 μM of inhibitor) that did not reduce OPC density (Fig. .1). The complex IV activity of mitochondria isolated from differentiated oligodendrocyte lineage cells persistently exposed to 1 μM and 10 μM of sodium azide for the 5 days decreased by 35.3% (mild) and 65.7% (moderate), respectively, compared with control samples when normalized using citrate synthase. The oxygen consumption of oligodendrocyte lineage cells was 80.1% and 66.1% following persistent inhibition with 1 μM and 10 μM of sodium azide, respectively, compared with controls. The number of OPCs in culture following persistent exposure to complex IV inhibitor was not significantly different compared with controls (Fig. .5a). There was a modest decrease in number of MBP+ cells with exposure to 10 μM of sodium azide (Fig. .5b). The morphological difference in oligodendrocytes differentiated with complex IV inhibitors was striking (Fig. .5c). Intense MBP immunofluorescence was noted in a number of oligodendrocytes without processes when complex IV was inhibited (Fig. .5g). The majority of (>90%) MBP+ cells extruded Trypan blue and did not express caspase 9 or show nuclear translocation of AIF (not shown). The findings on the percentage and morphology of MBP+ cells and OPC survival were confirmed using potassium cyanide (Supp. Info. Fig. 3).

To establish whether the reduction in percentage of MBP expressing cells that differentiated following constant exposure to a complex IV inhibitor was due to a reduction in the capacity to differentiate or differentiation into a more susceptible phenotype, we briefly exposed OPCs in suspension to inhibitors, prior to differentiation (Fig. .1). Inhibition of complex IV following brief exposure of cells to sodium azide, which is a reversible inhibitor of complex IV (Bennett et al., 1996; Berndt et al., 2001), was transient as indicated by spectrophotometric measurement of complex IV activity. The number of mature oligodendrocytes did not significantly change following transient complex IV inhibition (Fig. .5d,e). However, the morphological changes following brief exposure to complex IV inhibitor were as striking as the findings following persistent exposure (Fig. .5f).

## DISCUSSION

In this study, we identified mature oligodendrocytes differentiated from P0 rat as more prone to mitochondrial respiratory chain complex IV inhibitor mediated injury than OPCs. Furthermore, complex IV inhibition throughout and briefly prior to differentiation of OPCs impaired formation of oligodendrocyte processes.

Surprisingly inhibitors of mitochondrial respiratory chain complex IV did not significantly change the viability of cells that remained attached. Instead cells, mostly mature oligodendrocytes, detached from the slides following inhibition of complex IV in a concentration dependent manner. Energy requirements of mature oligodendrocyte processes appear to be much greater than processes of OPC. MMP in attached mature oligodendrocyte processes decreased significantly at lower levels of complex IV inhibition and to a greater extent as a proportion of controls than MMP in OPC processes. Furthermore, injury to processes as a prerequisite to cell detachment is suggested by the differentiation experiments with persistent inhibitors where process formation was impaired and cell detachment was modest. Imbalance of calcium downstream of mitochondrial injury may be important in the differential susceptibility of oligodendrocytes to complex IV inhibition (Marino et al., 2007; Micu et al., 2006).

Maturation dependent susceptibility of oligodendrocyte lineage cells has been the focus of a number of studies. Several studies have reported OPCs to be more vulnerable than MBP+ oligodendrocytes. Although mitochondria have been implicated in oligodendrocyte death in a number of studies complex IV activity was not determined in previous studies and mitochondrial activity and cell viability were assessed using MTT assay, which is based mitochondrial dehydrogenases. Superoxide, implicated in a number of previous studies, was not involved in the differential susceptibility in this study, which is not unexpected as MMP dissipation may prevent ROS upregulation and, unlike complex I, complex IV defects need to be severe to upregulate ROS (Adam-Vizi, 2005; Huttemann et al., 2008). A limitation of most cell culture studies is that only adherent cells are usually analyzed. We captured the detached cells following complex IV inhibition and detected expression of caspase 9 and nuclear translocation of AIF in majority of MBP+ cells (Garrido et al., 2006; Goldstein et al., 2000; Susin et al., 1999). Given the sparse expression of cell death markers in attached cells exposed to inhibitors, cell detachment per se may have triggered cell death. Investigation of differential susceptibility of oligodendrocytes to complex IV inhibitors *in vivo*, will address some of the limitations of *in vitro* studies. Furthermore, as the outcome of *in vitro* studies is influenced by differences in experimental paradigm, age of animals, species from which OPCs were derived and culture techniques our findings may not be applicable to oligodendrocytes differentiated *in vivo* and neonatal OPC from other species.

The most notable feature to be affected by complex IV inhibition during OPC differentiation was formation and structure of oligodendrocyte processes. Differentiation of OPCs into myelinating oligodendrocytes is a metabolically active process (Bauer et al., 2009; Cowell et al., 2008; Kirby et al., 2006; Kirischuk et al., 1995; Schoenfeld et al., 2009). Mitochondria provide acetyl-CoA, necessary for cholesterol synthesis and myelination (Schoenfeld et al., 2009). Mitochondrial location and activity appear to be important for calcium signaling (Haak et al., 2000; Simpson and Russell, 1996). The abundance of active mitochondria in the tip of oligodendrocyte processes and morphological differences following complex IV inhibition suggest an important role for mitochondria in process formation (Kirischuk et al., 1995). Oligodendrocyte lineage cells (O4+) lacked processes in a subset of PVL cases (Billiards et al., 2008; Segovia et al., 2008). A block in OPC differentiation has also been reported in MS (Chang et al., 2000, 2002; Kuhlmann et al., 2008; Wolswijk, 1998). ROS damage mtDNA within oligodendrocytes and enhancing DNA repair rescues oligodendrocytes, *in vitro* (Druzhyna et al., 2005). Further studies are needed to explore mitochondria and mitochondrial DNA within oligodendrocyte lineage cells in PVL and chronic MS lesions as well as determine whether remyelination is present in primary mtDNA disorders.

Mitochondrial dysfunction is now established in WMS and MS. Our *in vitro* findings recapitulate some of the features of WMS, PVL, and MS: distal oligodendrogliopathy involving first the processes and then leading to oligodendrocyte loss, partial preservation of OPCs, and differentiation block with limited remyelination. We propose complex IV defects as an important cause of oligodendrocyte injury and differentiation block of OPCs in demyelinating disorders.

## Abbreviations

CNS: central nervous system
COX: cytochrome c oxidase or complex IV of mitochondrial respiratory chain
COX-I: subunit-I of cytochrome c oxidase
MS: multiple sclerosis
mtDNA: mitochondrial DNA
OPCs: oligodendrocyte progenitor cells
PML: progressive multifocal leukoencephalopathy
PVL: periventricular leukomalacia
ROS: reactive oxygen species
WMS: white matter stroke

## Notes

Supporting Figure 1 Injury to oligodendrocyte lineage cells from P0 rat following complex IV inhibition by potassium cyanide. (A-C) Following exposure to potassium cyanide (KCN) there is a striking reduction in the number of cells (judged by nuclei stained with DAPI), derived from rodent mixed glial cultures, over 90℅ of which are oligodendrocyte lineage cells (a). There is a striking reduction in the percentage of cells expressing MBP following exposure to potassium cyanide (b). The number of NG2 expressing cells per field remains unchanged except with 100 μM KCN at 1 hour (c). KCN: potassium cyanide.

Supporting Figure 2 The measurement of mitochondrial superoxide production in live oligodendrocyte lineage cells from P0 rat. Oligodendrocyte lineage cells were analysed by flow cytometry for superoxide production following exposure to sodium azide (1 μM, 10 μM and 100 μM for 15 minutes, 3 and 36 hours). The extent of superoxide production by mitochondria was not significantly unaffected by complex IV inhibition compared with controls (10 μM and 100 μM for 36 hours and the overlap with controls shown). Superoxide production in OPCs did not significantly change following complex IV inhibition (data not shown). The experiments, controls and different concentrations of inhibitors, were performed on three occasions from different litters.

Supporting Figure 3 Differentiation of P0 rat OPCs persistently exposed to sublethal concentrations of potassium cyanide, a complex IV inhibitor. (A-C) The total number of cells remaining in chamber slides, judged using DAPI staining, following 5 days of differentiation whilst exposed to different sublethal concentrations of potassium azide is similar to controls (a). Interestingly, the percentage of MBP+ cells did not significantly change when differentiated with KCN concentrations that did not reduce the density of OPCs (b) However, the morphology of MBP expressing cells exposed to the inhibitor is different from controls, with over twice as many MBP reactive cells lacking processes (c). The oxygen consumption of oligodendrocyte lineage cells was 81.9℅ and 63.3℅ following persistent inhibition with 1 μM and 10 μM of potassium cyanide, respectively. The experiments, controls and different concentrations of inhibitors in triplicate chambers, were performed on six occasions from different litters. Based on morphology, mature oligodendrocytes were categorised into rounded (category I shown in G I), simple processes (category II shown in G II), complex processes (category III) or with MBP+ membrane (category IV).
