# Supplementary figures and images for: Injury and differentiation following inhibition of mitochondrial respiratory chain complex IV in rat oligodendrocytes
Source: Glia. 2010 Nov 15;58(15):1827–37. doi: 10.1002/glia.21052 (PMC3580049; doi:10.1002/glia.21052)

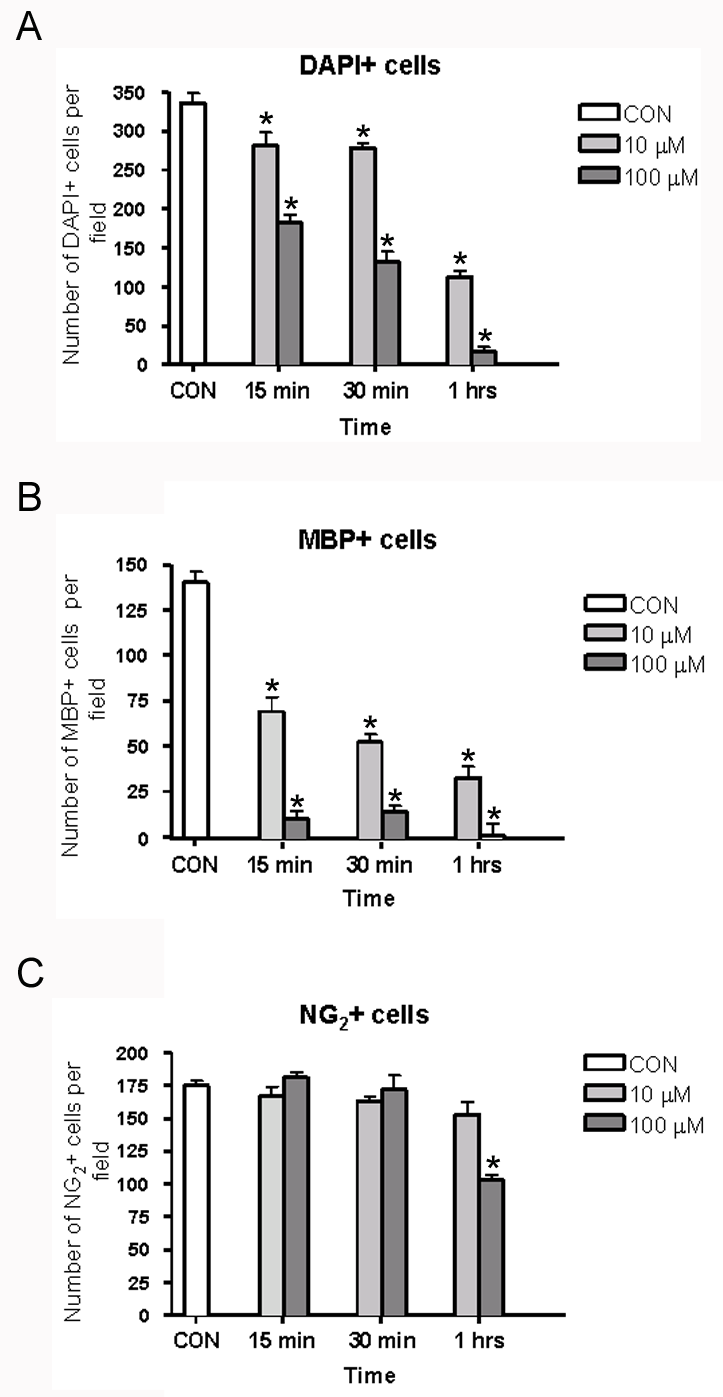

Supplement: Supporting Figure 1 — Injury to oligodendrocyte lineage cells from P0 rat following complex IV inhibition by potassium cyanide. (A-C) Following exposure to potassium cyanide (KCN) there is a striking reduction in the number of cells (judged by nuclei stained with DAPI), derived from rodent mixed glial cultures, over 90℅ of which are oligodendrocyte lineage cells (a). There is a striking reduction in the percentage of cells expressing MBP following exposure to potassium cyanide (b). The number of NG2 expressing cells per field remains unchanged except with 100 μM KCN at 1 hour (c). KCN: potassium cyanide. [file glia0058-1827-sd1.tif]

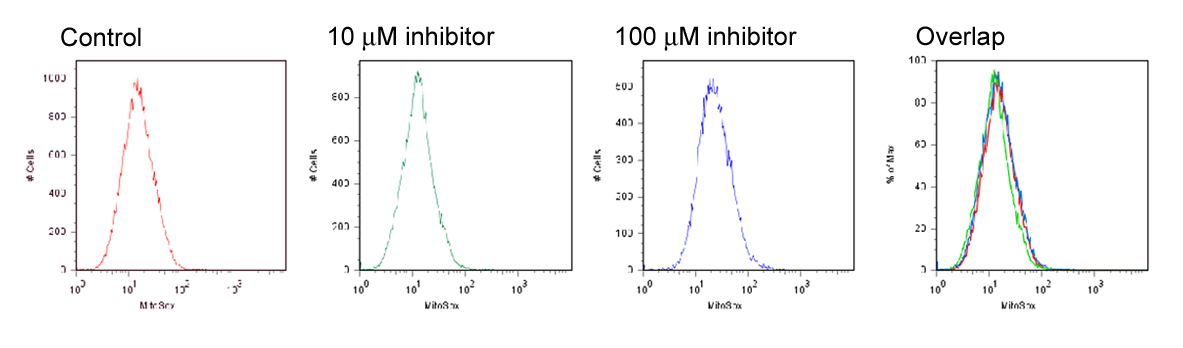

Supplement: Supporting Figure 2 — The measurement of mitochondrial superoxide production in live oligodendrocyte lineage cells from P0 rat. Oligodendrocyte lineage cells were analysed by flow cytometry for superoxide production following exposure to sodium azide (1 μM, 10 μM and 100 μM for 15 minutes, 3 and 36 hours). The extent of superoxide production by mitochondria was not significantly unaffected by complex IV inhibition compared with controls (10 μM and 100 μM for 36 hours and the overlap with controls shown). Superoxide production in OPCs did not significantly change following complex IV inhibition (data not shown). The experiments, controls and different concentrations of inhibitors, were performed on three occasions from different litters. [file glia0058-1827-sd2.tif]

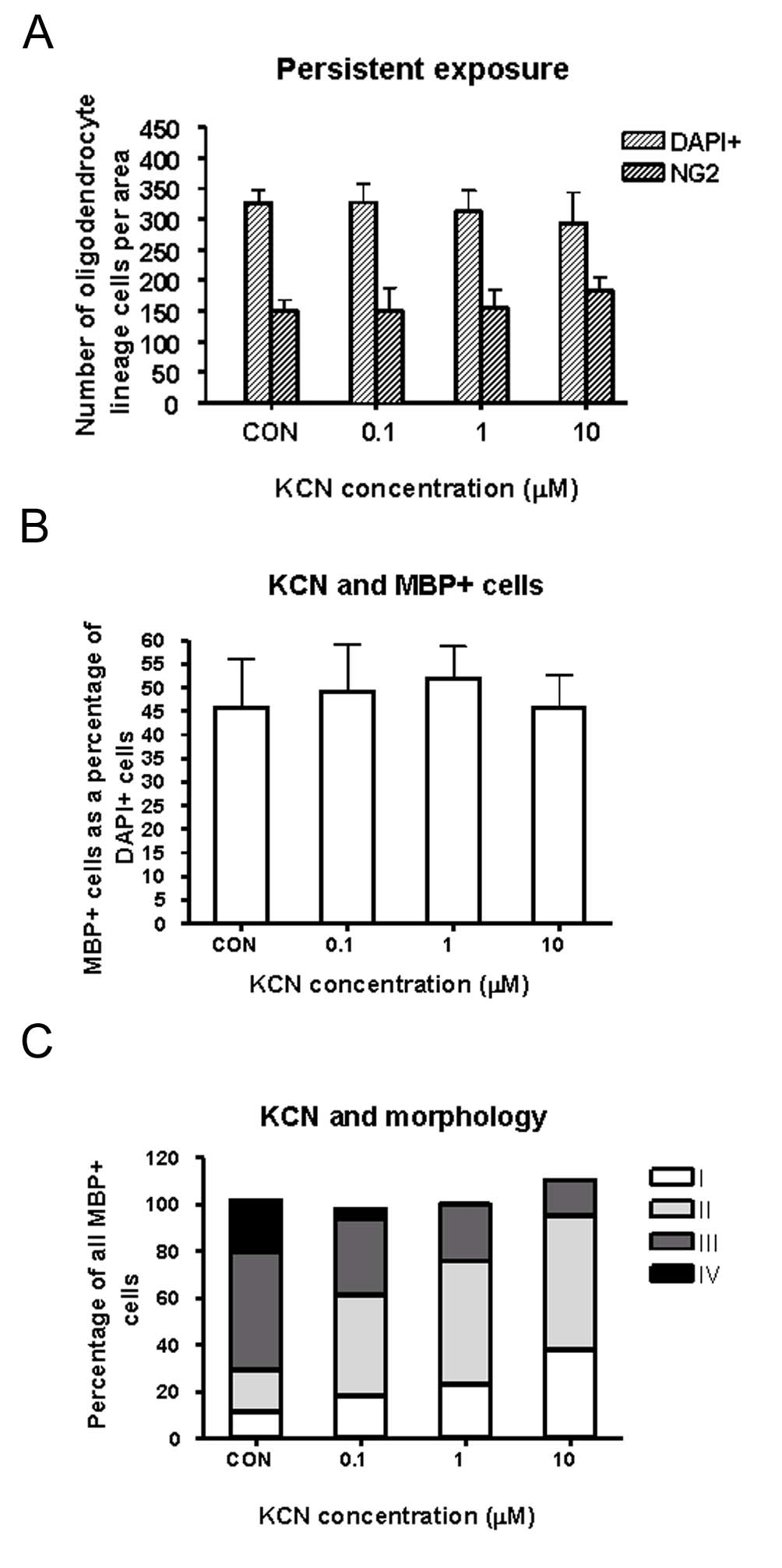

Supplement: Supporting Figure 3 — Differentiation of P0 rat OPCs persistently exposed to sublethal concentrations of potassium cyanide, a complex IV inhibitor. (A-C) The total number of cells remaining in chamber slides, judged using DAPI staining, following 5 days of differentiation whilst exposed to different sublethal concentrations of potassium azide is similar to controls (a). Interestingly, the percentage of MBP+ cells did not significantly change when differentiated with KCN concentrations that did not reduce the density of OPCs (b) However, the morphology of MBP expressing cells exposed to the inhibitor is different from controls, with over twice as many MBP reactive cells lacking processes (c). The oxygen consumption of oligodendrocyte lineage cells was 81.9℅ and 63.3℅ following persistent inhibition with 1 μM and 10 μM of potassium cyanide, respectively. The experiments, controls and different concentrations of inhibitors in triplicate chambers, were performed on six occasions from different litters. Based on morphology, mature oligodendrocytes were categorised into rounded (category I shown in G I), simple processes (category II shown in G II), complex processes (category III) or with MBP+ membrane (category IV). [file glia0058-1827-sd3.tif]
