# Supplementary material for: Injury and differentiation following inhibition of mitochondrial respiratory chain complex IV in rat oligodendrocytes
Source: Glia. 2010 Nov 15;58(15):1827–37. doi: 10.1002/glia.21052 (PMC3580049; doi:10.1002/glia.21052)
Supplement: Supplementary file 4 [file glia0058-1827-sd4.doc]

**Supporting Information Table 1.**

| Report | Type of Injury | OPCs | Differentiating or mature oligodendrocytes | Length of differentiation  (days) |
| --- | --- | --- | --- | --- |
| (Andrews et al. 1998)  (rat and CG4 *in vitro*) | TNFa and IFNg | No markers  (X) | NA | 3 |
| (Back et al. 1998)  (rat *in vitro*) | Glutathione depletion | O4^+^O1^-^  (X) | MBP^+^ | 8 |
| (McDonald et al. 1998) (mouse *in vitro*) | Kainate | A2B5^+^  (X) | O1^+^MBP^+^ | 14 |
| (Scurlock and Dawson 1999) (HOG and rat *in vitro*) | TNFa, IFNg  and ceramide | O2A^+^  (X) | No marker | 21 (HOG) |
| (Almazan et al. 2000)  (rat *in vitro*) | Cadmium | A2B5+GalC-  (X) | A2B5^-^GalC^+^ | 9 |
| (Fern and Moller 2000) (rat *in vitro*) | Hypoxia-Ischemia (OGD) | O4^+^GalC^-^  (X) | A2B5^-^O4^-^GalC^+^ | 30-60 |
| (Itoh et al. 2000)  (rat *in vitro*) | AMPA receptor activation | A2B5^+^GalC^-^  vs.  A2B5^+^GalC^+^  MBP^-^  (X) | NA | 4 |
| (Back et al. 2002)  (rat *in vitro*) | Hypoxia- ischemia | O4^+^O1^+^  vs.  O4^+^O1^-^  (X) | No marker (morphology) | P2 and P7 |
| (Khorchid et al. 2002)  (rat *in vitro*) | Catecholamine | A2B5^+^  (X) | GalC^+^CNPase^+^ MBP^+^ | 12 |
| (Rosenberg et al. 2003)  (rat *in vitro*) | Kainate | A2B5^+^O4^+^O1^+^  MBP^-^  (X) | MBP^+^ | 14 |
| (Baud et al. 2004a)  (rat *in vitro*) | H2O2 | A2B5^+^O4^+^O1^-^MBP^-^  (X) | MBP^+^ | 14 |
| (Baud et al. 2004b)  (rat *in vitro*) | Cystine deprivation | A2B5^+^O4^+^O1^-^MBP^-^  (X) | MBP^+^ | 14 |
| (Fragoso et al. 2004)  (rat *in vitro*) | H2O2 | A2B5^+^  (X) | MBP^+^ | 12 |
| Pirianov, 2006  (CG-4 *in vitro*)  (Pirianov et al. 2006) | Staurosporine and ceramide | Undifferentiated (X)  No marker | Differentiated | Not stated |
| (Miller et al. 2007)  (rat *in vitro*) | LPS and microglia | A2B5^+^NG2^+^GalC^+^MBP^-^  (X) | A2B5^-^NG2^-^GalC^-^MBP^+^ | 3 |
| (Gerstner et al. 2008)  (rat *in vivo* and *in vitro*) | Hyperoxia | O4^+^O1^-^MBP^-^  (X) | MBP^+^ | 7 |
| (Schoenfeld et al. 2009) (HOG *in vitro*) | Rotenone  (complex I inhibitor) | undifferentiated  vs.  differentiated for 1 day (X)  (no markers) | Differentiated for 10 days  (no markers) | 10 |

(X): indicates the phenotype with greatest susceptibility.
