# Supplementary material for: Injury and differentiation following inhibition of mitochondrial respiratory chain complex IV in rat oligodendrocytes
Source: Glia. 2010 Nov 15;58(15):1827–37. doi: 10.1002/glia.21052 (PMC3580049; doi:10.1002/glia.21052)
Supplement: Supplementary file 5 [file glia0058-1827-sd5.doc]

**Supporting Information Table 2. Proliferation and migration of OPCs following exposure to complex IV inhibitor.**

|  | Control | 1 μM | 10 μM | 100 μM |
| --- | --- | --- | --- | --- |
| Proliferation  (Olig2^+^ cells/field) | 105.52  ± 9.62 | 106.31  ± 5.08 | 118.61  ± 13.12 | 98.82  ± 5.10 |

For proliferation, OPCs were exposed to PDGF and FGF with or without sodium azide for 48 hours.

Means ± Standard Deviation.
